# Fuji-SfM dataset: A collection of annotated images and point clouds for Fuji apple detection and location using structure-from-motion photogrammetry

**DOI:** 10.1016/j.dib.2020.105591

**Published:** 2020-04-21

**Authors:** Jordi Gené-Mola, Ricardo Sanz-Cortiella, Joan R. Rosell-Polo, Josep-Ramon Morros, Javier Ruiz-Hidalgo, Verónica Vilaplana, Eduard Gregorio

**Affiliations:** a Research Group in AgroICT & Precision Agriculture, Department of Agricultural and Forest Engineering, Universitat de Lleida (UdL) – Agrotecnio Center, Lleida, Catalonia, Spain; b Department of Signal Theory and Communications, Universitat Politècnica de Catalunya, Barcelona, Catalonia, Spain

**Keywords:** Fruit detection, Yield prediction, Yield mapping, Structure-from-motion, Photogrammetry, Mask R-CNN, Terrestrial remote sensing

## Abstract

The present dataset contains colour images acquired in a commercial Fuji apple orchard (*Malus domestica* Borkh. cv. Fuji) to reconstruct the 3D model of 11 trees by using structure-from-motion (SfM) photogrammetry. The data provided in this article is related to the research article entitled “Fruit detection and 3D location using instance segmentation neural networks and structure-from-motion photogrammetry” [1]. The Fuji-SfM dataset includes: (1) a set of 288 colour images and the corresponding annotations (apples segmentation masks) for training instance segmentation neural networks such as Mask-RCNN; (2) a set of 582 images defining a motion sequence of the scene which was used to generate the 3D model of 11 Fuji apple trees containing 1455 apples by using SfM; (3) the 3D point cloud of the scanned scene with the corresponding apple positions ground truth in global coordinates. With that, this is the first dataset for fruit detection containing images acquired in a motion sequence to build the 3D model of the scanned trees with SfM and including the corresponding 2D and 3D apple location annotations. This data allows the development, training, and test of fruit detection algorithms either based on RGB images, on coloured point clouds or on the combination of both types of data.

Specifications table

**Table:** 

Subject	Agronomy and Crop Science, Horticulture, Computer Vision and Pattern Recognition
Specific subject area	Precision Agriculture, Fruit Detection, Remote Sensing, Machine Learning, Deep Learning, Agrobotics
Type of data	RGB colour images
	Instance segmentation binary masks
	Motion sequence images
	3D coloured point clouds
	3D fruit location annotations
How data were acquired	Images were taken freehand, using an EOS 60D DSLR Canon camera with an 18 MP (5184 × 3456 px) CMOS APS-C sensor (22.3 × 14.8mm), and a Canon EF-S 24mm f/2.8 STM lens.
Data format	Raw colour images: *JPG*
	Instance segmentation binary masks: *CSV* and *JSON*
	3D coloured point clouds: *TXT*
	3D fruit location annotations: *TXT*
Parameters for data collection	The camera focal length was 35 mm (38mm film equivalent focal length), which corresponded to a field of view of [59° 10’, 50° 35’] (horizontal, vertical). Images were taken from a distance of 3m between the camera and the middle plane of the row. The vertical and horizontal overlapping between neighbouring images was higher than 30% and 90%, respectively.
Description of data collection	A total of 11 Fuji apple trees containing 1455 apples were photographed from 53 position (per side) distributed along the row of trees, having a separation of approximately 22 cm between two consecutive positions. In each position, a vertical sweep of 5-6 images was practiced, obtaining images of all tree heights -from the soil/trunk to the upper part of the trees-. The East side of the row of trees was photographed in the morning, while the West face in the afternoon, obtaining a similar illumination conditions in both faces.
Data source location	City/Town/Region: Agramunt, Catalonia
	Country: Spain
	GPS coordinates for collected data: E: 336297 m, N: 4623494 m, 312 m a.s.l., UTM 31T - ETRS89
Data accessibility	Repository name:*Zenodo*
	Data identification:*Fuji-SfM dataset*
	DOI:https://doi.org/10.5281/zenodo.3712808
	Direct URL to data:https://zenodo.org/record/3712808#.XnD82iNCe01
Related research article	[1] Gené-Mola J, Sanz-Cortiella R, Rosell-Polo JR, Morros J-R, Ruiz-Hidalgo J, Vilaplana V, Gregorio E. 2019.
	Fruit detection and 3D location using instance segmentation neural networks and structure-from-motion photogrammetry. (Submitted)

## Value of the data

This data is useful for the research community for the following reasons:

• First dataset for fruit detection with 3D coloured point clouds generated by applying structure-from-motion photogrammetry. This dataset differs from other existing fruit detection datasets based on RGB, RGB-D and LiDAR sensors [2], [3], [4] by providing 3D point clouds that were obtained with SfM. Furthermore, it includes the set of motion sequence images used for point cloud generation and a set images manually annotated with instance segmentation masks.
• Computer vision community can benefit from these data to test new object detection and segmentation algorithms either based on 2D or on 3D data.
• Annotations provided can be used for training machine learning systems used in agriculture with applications such as yield prediction, yield mapping and automated harvesting [5], [6], [7], [8].
• Finally, a further significant value of this data is that it provides for the first time a very detailed annotated dataset for benchmarking fruit detection based on machine learning and 3D computer vision techniques.
• This dataset allows to reproduce all methods and results reported in the corresponding research paper [1].

## 1 Data

**Fig. 1:**
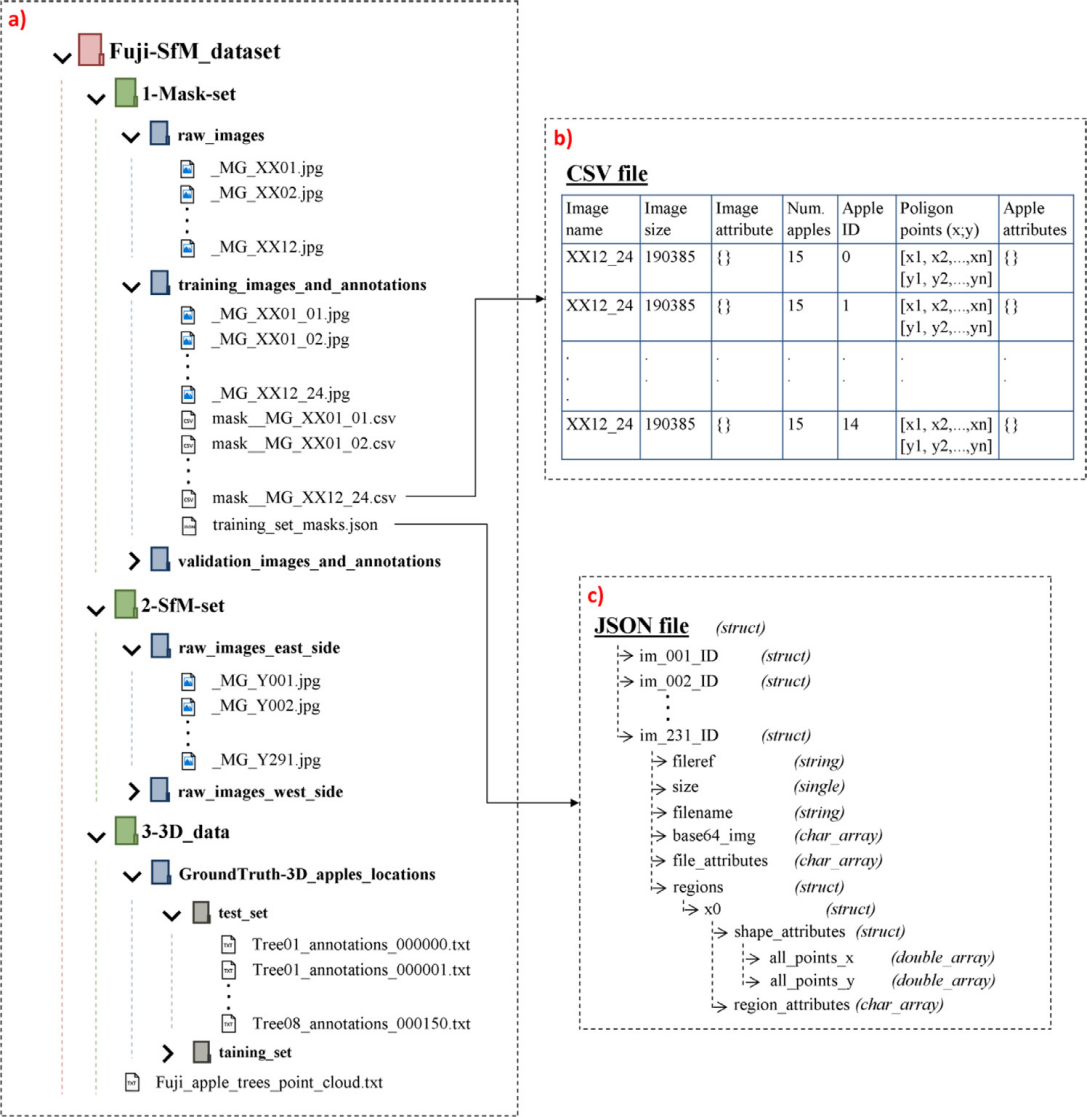
Illustration of the dataset structure once it is unpacked. a) dataset structure; b) CSV files description; c) JSON files structure.

The Fuji-SfM dataset includes annotated data for 2D and 3D fruit detection. This dataset can be downloaded at http://doi.org/10.5281/zenodo.3712808 [9]. Once the dataset is unpacked, data is organized as shown in Fig. 1.

**Fig. 2:**
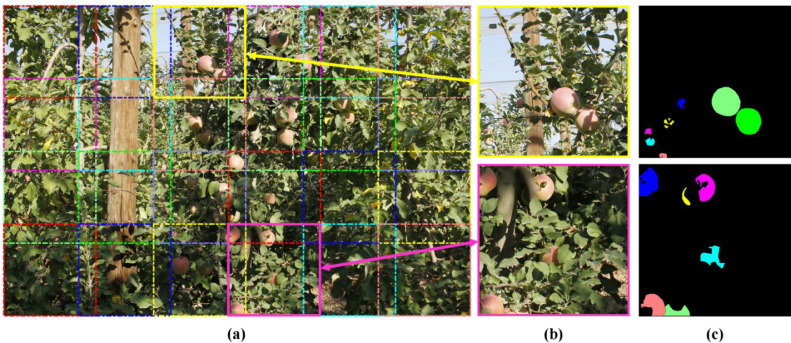
Illustration of an image from the *Mask-set*. a) Image cropping borders. b) Example of two sub-images. c) Ground truth segmentation masks.

The *1-Mask-set* folder includes 12 raw images of Fuji apple trees. This set of images was used in [1] to train and validate the Mask-RCNN [10]. Since the performance of object detection and segmentation neural networks decreases when detecting small objects [11], each *Mask-set* image was divided into 24 sub-images of 1024 × 1024 px (Fig. 2a and Fig. 2b). The resulting 288 sub-images were split in training (231 sub-images) and validation (57 sub-images) sub-sets and were manually annotated generating the apples segmentation masks ground truth (Fig. 2c). Image annotations were saved in CSV and JSON file formats. Fig. 1(b and c) details the structure of CSV and JSON files, respectively. From this files, the ground truth mask of an apple *i* can be obtained as a set of polygon points (x, y), either from the (i+1) row, 6th column of the CSV file, or from the JSON structure *JSON_file.img_ID.regions.x0.shape_attributes*. Then, to convert the set of polygon points into a binary mask, one could use the MATLAB® (Math Works Inc., Natick, Massachusetts, USA) function *poly2mask*, or equivalent functions for other programming languages.

**Fig. 3:**
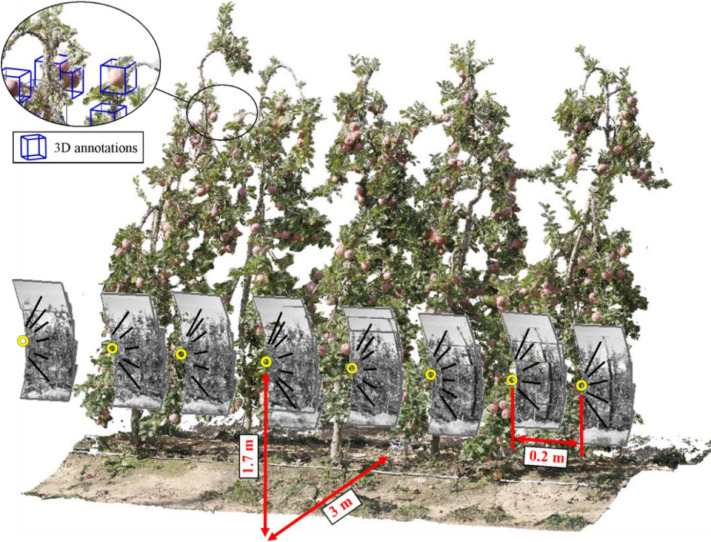
Isometric view of five scanned trees and illustration of the photographic process layout. Yellow circles show the photographic position. Blue 3D rectangular bounding boxes illustrate the apple position ground truth annotations.

The *2-SfM-set* folder includes 582 raw images (291 per row of trees side) of 11 consecutive Fuji apple trees. This set of images was used to generate the 3D model of the scanned scene by applying structure-from-motion (SfM) photogrammetry. The obtained 3D model was georeferenced in global world coordinates and saved as a point cloud in TXT format: *\Fuji-SfM_dataset\3-3D_data\Fuji_apple_trees_point_cloud.txt*. Each row of this point cloud file correspond to a single 3D point, giving the information of [x, y, z, R, G, B], where, [x, y, z] is the point position in global coordinates, and [R,G,B] is the point colour with 8-bit precision values ranging from 0 to 255. The point cloud was manually labelled by placing 3D rectangular bounding boxes around each apple position (blue bounding boxes illustrated in Fig. 3). A total of 1455 apples were annotated. Each fruit location annotation was saved in a TXT file where the first row corresponds to the position [x, y, z] of the apple centre, while the following eight rows indicate the positions of the bounding box corners.

## 2 Experimental design, materials, and methods

Images provided in Fuji-SfM dataset were acquired on September 2017 in a commercial Fuji apple orchard located in Agramunt, Catalonia, Spain (E: 336,297 m; N: 4,623,494 m; 312 m a.s.l., UTM 31T - ETRS89). The scanned trees were trained in a tall spindle system, with a maximum canopy height of 3.5m and width of 1.5 m, approximately. *Mask-set* images were taken from different randomly selected zones of the orchard, while *SfM-set* images were acquired from both sides of 11 consecutive trees containing a total of 1455 apples. All data was acquired three weeks before harvesting, at BBCH phenological growth stage 85 [12].

The camera used for data acquisition was an EOS 60D DSLR Canon camera (Canon Inc. Tokyo, Japan), with an 18 MP (5184 × 3456 px) CMOS APS-C sensor (22.3 × 14.9 mm), and a Canon EF-S 24mm f/2.8 STM lens (35 mm film equivalent focal length of 38 mm). All images were taken freehand from a distance of approximately 3 m from the trees centre, and at a height of 1.7 m (Fig. 3). Images from the east side of the row of trees were photographed in the morning (11:53 – 12:26h), while the west side was photographed in the afternoon (15:27 – 16:05h) under natural illumination conditions.

Fig. 3 illustrates the data acquisition process followed for the *SfM-set*. Yellow circles represent the camera centre of different photographic positions. The separation between two consecutive positions was 0.2 m, corresponding to a total of 53 photographic positions per row of trees side. From each camera position, a vertical sweep of 5-6 photographs was taken (black lines). With this configuration, a total of 291 images were taken per side, with a vertical/horizontal overlapping between neighbouring images higher than a 30/90 %, respectively (as shown in [1], Fig. 2).

*SfM-set* images were used to reconstruct the 3D model of the 11 scanned trees. A multi-view structure-from-motion photogrammetry based on bundle adjustment [13] was applied to generate the 3D point cloud of each side of the row of trees. This 3D model generation was carried out using Agisoft Professional Photoscan software (v1.4, Agisoft LLC, St. Petersburg, Russia). A set of known markers in the scene was used to scale and georeferencing the obtained point clouds. Then, point clouds from both sides of the row of trees were merged, obtaining a complete representation of the scanned trees in a single point cloud.

*Mask-set* images were manually labelled with apple segmentation masks, allowing the use of this set of images to train and test 2D instance segmentation algorithms. This annotation was performed using the VIA annotation software [14], enclosing individual apples with polygon region shapes. The point cloud of the 11 scanned trees was also manually labelled. Similarly than in [15], the 3D annotation was carried out using the software CloudCompare (Cloud Compare [GPL software] v2.9 Omnia), placing 3D rectangular bounding boxes around each apple, as can be seen in the zoomed-in region of Fig. 3.

## References

[1] Gené-Mola J., Sanz-Cortiella R., Rosell-Polo J.R., Morros J.-R., Ruiz-Hidalgo J., Vilaplana V., Gregorio E. (2020). Fruit detection and 3D location using instance segmentation neural networks and structure-from-motion photogrammetry. Comput. Electron. Agric..

[2] Oltean M. (2018). Fruits 360 dataset. Mendeley Data.

[3] Gené-Mola J., Vilaplana V., Rosell-Polo J.R., Morros J.-R., Ruiz-Hidalgo J., Gregorio E. (2019). KFuji RGB-DS database: Fuji apple multi-modal images for fruit detection with color, depth and range-corrected IR data. Data Br..

[4] Gené-Mola J., Gregorio E., Auat Cheein F., Guevara J., Llorens J., Sanz-Cortiella R., Escolà A., Rosell-Polo J.R. (2020). LFuji-air dataset: Annotated 3D LiDAR point clouds of Fuji apple trees for fruit detection scanned under different forced air flow conditions. Data Br..

[5] Koirala A., Walsh K.B., Wang Z., McCarthy C. (2019). Deep learning – Method overview and review of use for fruit detection and yield estimation. Comput. Electron. Agric..

[6] Gongal A., Amatya S., Karkee M., Zhang Q., Lewis K. (2015). Sensors and systems for fruit detection and localization: A review. Comput. Electron. Agric..

[7] Gené-Mola J., Gregorio E., Auat Cheein F., Guevara J., Llorens J., Sanz-Cortiellaa R., Escolà A., Rosell-Polo J.R. (2019). Fruit detection, yield prediction and canopy geometric characterization using LiDAR with forced air flow. Comput. Electron. Agric..

[8] Bac C.W., Van Henten E.J., Hemming J., Edan Y. (2014). Harvesting Robots for High-value Crops: State-of-the-art Review and Challenges Ahead. J. F. Robot.

[9] Gene-Mola J., Sanz-Cortiella R., Rosell-Polo J.R., Morros J.-R., Ruiz-Hidalgo J., Vilaplana V., Gregorio E. (2020). Fuji-SfM dataset. Zenodo.

[10] He K., Gkioxari G., Dollar P., Girshick R. (2017). Mask RCNN. Proc. IEEE Int. Conf. Comput. Vis. 2017.

[11] Gené-Mola J., Vilaplana V., Rosell-Polo J.R., Morros J.R., Ruiz-Hidalgo J., Gregorio E. (2019). Multi-modal deep learning for Fuji apple detection using RGB-D cameras and their radiometric capabilities. Comput. Electron. Agric..

[12] U. Meier, Growth stages of mono- and dicotyledonous plants, 2001. doi:10.5073/bbch0515.

[13] Triggs B., McLauchlan P.F., Hartley R.I., Fitzgibbon A.W. (2000). Bundle Adjustment — A Modern Synthesis Vision Algorithms: Theory and Practice. Vis. Algorithms Theory Pract..

[14] Dutta A., Zisserman A. (2019). The VIA Annotation Software for Images, Audio and Video. Proc. 27th ACM Int. Conf. Multimed..

[15] Gené-Mola J., Gregorio E., Guevara J., Auat F., Sanz-cortiella R., Escolà A., Llorens J., Morros J.-R., Ruiz-Hidalgo J., Vilaplana V., Rosell-Polo J.R. (2019). Fruit detection in an apple orchard using a mobile terrestrial laser scanner. Biosyst. Eng..

